# Anti-cancer mechanism of traditional Chinese medicine natural products targeting ferroptosis

**DOI:** 10.1186/s13020-026-01363-7

**Published:** 2026-03-16

**Authors:** Jiying Zhou, Peiying Lu, Meiling Guo, Xiaodong Chen, Keyan Chai, Haojia Wang, Lijia Zhou, Yiyan Zhai, Jiaqi Li, Chuanqi Qiao, Siyun Yang, Hua Luo, Peizhi Ye, Jiarui Wu

**Affiliations:** 1 School of Chinese Materia Medica, Beijing University of Chinese Medicine, Beijing, 102488 China; 2 Guangxi University of Chinese Medicine, Nanning, 530001 China; 3 National Cancer Center / National Clinical Research Center for Cancer, Chinese Medicine Department of the Cancer Hospital of the Chinese Academy of Medical Sciences and Peking Union Medical College, Beijing, 100021 China

**Keywords:** Traditional Chinese medicine, Natural products, Ferroptosis, Mechanism, Anti-tumor

## Abstract

Ferroptosis is a novel iron-dependent form of programmed cell death characterized by the accumulation of lipid peroxides. Its mechanism involves the disruption of iron metabolism, imbalances in the antioxidant system, and lipid peroxidation. As a leading global cause of death, cancer treatment often faces challenges such as drug resistance and adverse side effects. Traditional Chinese medicine (TCM), as a complementary and alternative therapy, demonstrates significant potential in tumor treatment due to its multi-targeted and multi-pathway regulatory advantages. Recent studies reveal that numerous natural products in Chinese herbal medicines can inhibit tumor growth by inducing ferroptosis. This review systematically elucidates the core molecular mechanisms of ferroptosis, encompassing iron metabolism, lipid peroxidation, and various regulatory pathways. It highlights the research progress on how natural products from TCM induce ferroptosis by regulating key targets in lung cancer, breast cancer, colorectal cancer, gastric cancer, and liver cancer. This review aims to provide a reference for the development of anticancer drugs based on TCM.

**Graphical Abstract:**

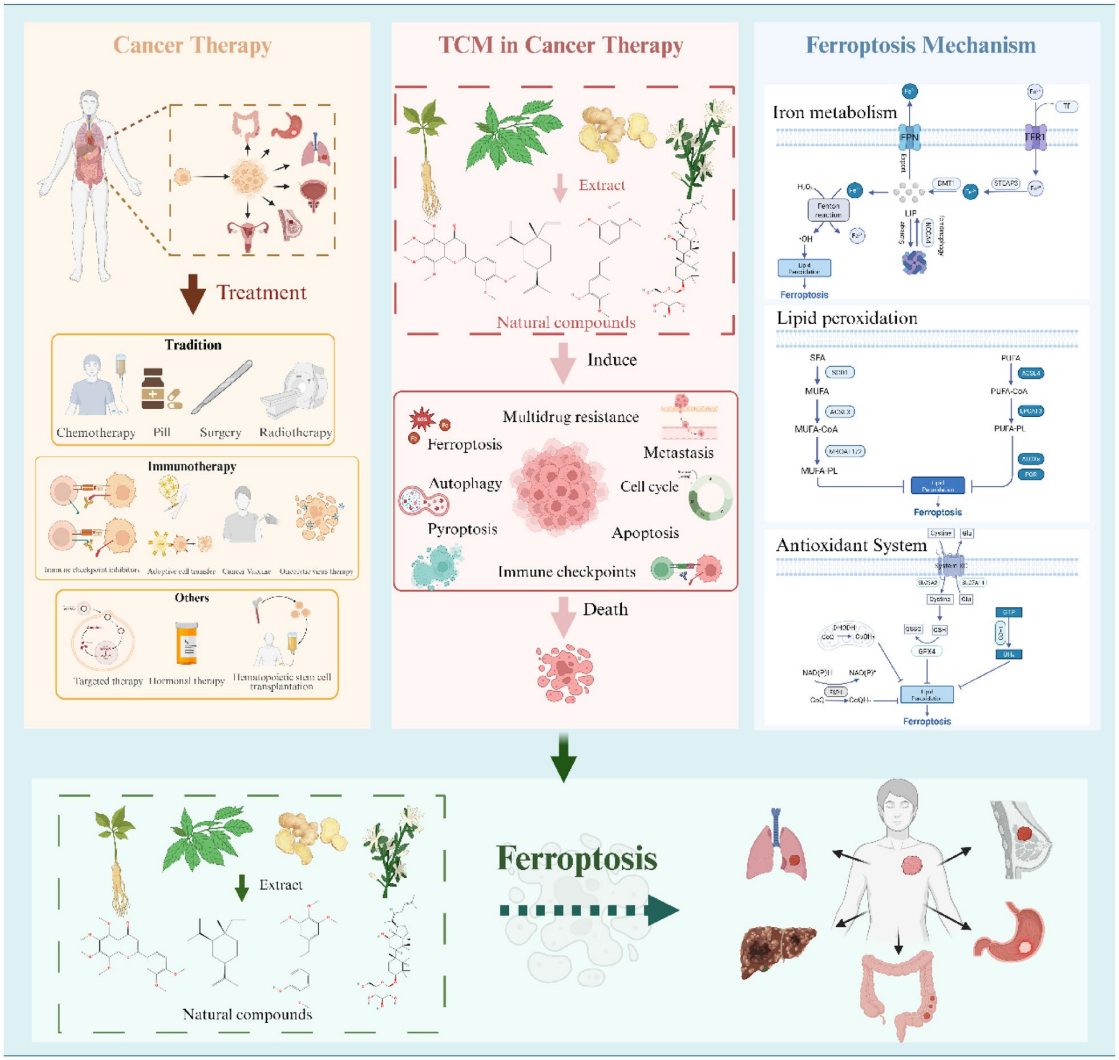

## Introduction

Cell death is a complex and interconnected process within organisms, playing a crucial role in maintaining tissue homeostasis and preventing diseases. Ferroptosis, a current hotspot in tumor research, was first proposed by Dixon in 2012 as a mode of iron-dependent non-apoptotic cell death characterized by the accumulation of lipid reactive oxygen species (ROS) [1]. The mechanisms of ferroptosis are primarily associated with disturbances in iron metabolism, imbalances in the amino acid antioxidant system, and the accumulation of lipid peroxides, which are distinctly different from other forms of cell death, such as necrosis, apoptosis, autophagy, and pyroptosis. Its main features include significant mitochondrial shrinkage, increased mitochondrial membrane density, reduced or absent mitochondrial cristae, rupture of the outer mitochondrial membrane, and no apparent changes in nuclear morphology [2, 3]. Ferroptosis is a unique and important form of programmed cell death that plays a role in various physiological processes and disease states, including ischemia, cancer, and neurodegeneration [4, 5]. Therefore, in-depth research into the mechanisms of ferroptosis is expected to provide new theoretical foundations for treatment strategies related to it and associated diseases.

Cancer is a significant public health issue, being a leading cause of death in every country and a major barrier to increased life expectancy, as well as a crucial factor contributing to the global disease burden. In 2022, nearly 20 million new cancer cases were reported worldwide, and it is projected that by 2050, the incidence of cancer will reach 35 million [6]. Estimates suggest that approximately one in five men or women will be diagnosed with cancer in their lifetime, while about one in nine men and one in twelve women will die from the disease. In the fight against tumors, various anti-tumor drugs and technologies have been developed, achieving numerous successes. However, cancer treatment remains a significant challenge, and the development of more precise, effective, and efficient therapeutic methods for tumors has been a global concern [7]. Ferroptosis, as a unique mechanism of cell death, may offer a promising new approach for cancer treatment [8, 9].

Current cancer treatment modalities, including surgery, radiotherapy, chemotherapy, targeted therapy, and immunotherapy [10], have significantly improved patient outcomes. However, challenges such as adverse effects and the development of therapeutic resistance remain important clinical limitations [11, 12]. In this context, natural products derived from TCM have attracted increasing research interest as a potential source of complementary therapeutic agents [13, 14]. Emerging evidence suggests that certain TCM-derived compounds may exert anti-tumor effects through multiple mechanisms, including the modulation of cell death pathways such as apoptosis, autophagy, and ferroptosis, as well as the regulation of angiogenesis, oncogene expression, and cell cycle progression [15]. Furthermore, preclinical studies have indicated that some of these compounds may alleviate chemotherapy-induced toxicity or modulate immune responses, although their clinical efficacy, safety profiles, and mechanisms of action require further systematic investigation.

Building on this empirical foundation, an increasing number of studies indicate that traditional herbal medicine can inhibit the growth of malignant tumors by inducing ferroptosis in cancer cells. Unlike conventional therapies, these products can selectively trigger this iron-dependent form of cell death by targeting key ferroptosis pathways: they may dysregulate iron metabolism to increase intracellular labile iron, inhibit antioxidant defense systems, or promote lipid peroxidation. Leveraging these mechanisms offers a promising strategy to enhance tumor sensitivity to standard therapies like chemotherapy and radiotherapy, and to overcome drug resistance. Resveratrol induces ferroptosis in triple-negative breast cancer cells via NEDD4L-mediated ubiquitination and degradation of GPX4 [16]. Notably, artesunate has been shown to synergize with sorafenib to promote ferroptosis in hepatocellular carcinoma, demonstrating its potential to overcome therapeutic resistance [17]. Therefore, targeting ferroptosis represents a pivotal mechanism through which TCM natural products exert anti-tumor effects. This review focuses on the natural products of TCM, summarizing the current research status on the induction of ferroptosis in common cancers such as lung cancer, breast cancer, and colorectal cancer, and analyzing their regulatory patterns on cancer-related ferroptosis signaling pathways, aiming to provide a reference for the development of anti-tumor drugs in TCM.

## Mechanisms of ferroptosis

**Fig. 1:**
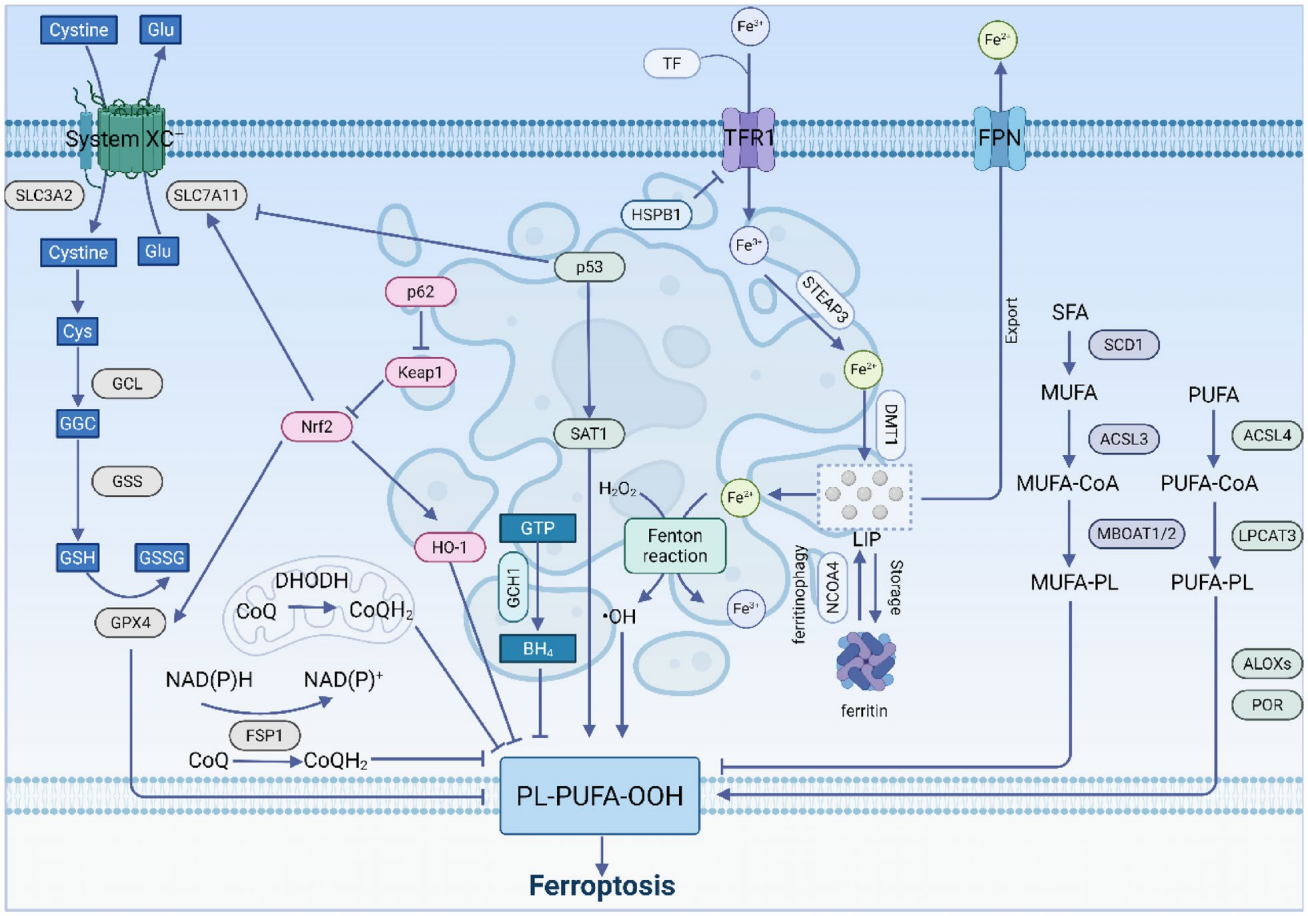
Schematic diagram of the mechanism of ferroptosis

The occurrence of ferroptosis is closely related to disruptions in iron metabolism, imbalances in the amino acid antioxidant system, and the accumulation of lipid peroxides within cellular metabolic regulatory systems [18]. Disruption of iron metabolism leads to the accumulation of free iron, which triggers lipid peroxidation of polyunsaturated fatty acids (PUFAs) through the Fenton reaction. Meanwhile, key antioxidant systems such as GPX4/GSH and FSP1/CoQ_10_ become ineffective, failing to timely eliminate peroxidation products, ultimately resulting in cell membrane damage and cell death [19] (Fig. 1).

### Iron metabolism

Iron metabolism regulation is a core mechanism driving ferroptosis. Essentially, it involves the disruption of the dynamic balance of cellular iron uptake, storage, utilization, and efflux, resulting in the excessive accumulation of redox-active free Fe^2^⁺ within the cell. This accumulation leads to the generation of ROS through the Fenton reaction, ultimately triggering lethal lipid peroxidation. Extracellular ferric iron binds to transferrin (TF) to form the TF-Fe^3^⁺ complex, which is internalized into the cell via transferrin receptor 1(TFR1)-mediated endocytosis [20]. Within the endosome, STEAP3 reduces Fe^3^⁺ to Fe^2^⁺. DMT1 then releases Fe^2^⁺ into the cytosolic labile iron pool (LIP). Excess Fe^2^⁺ can either be exported out of the cell via ferroportin (FPN) or oxidized to Fe^3^⁺ and stored in ferritin to maintain iron homeostasis. Ferritin is composed of heavy chain (FTH1) and light chain (FTL), and can be degraded through autophagy, catalyzed by Nuclear receptor coactivator 4 (NCOA4), leading to the release of Fe^2^⁺ from ferritin after reduction from Fe^3^⁺, entering the LIP [21, 22]. When iron homeostasis is disrupted, excess Fe^2^⁺ generates a large amount of ROS through the Fenton reaction, which reacts with PUFAs in the cell membrane and plasma membrane, producing phospholipid hydroperoxides and ultimately resulting in cellular ferroptosis [18].

### Lipid peroxidation

Another key feature of ferroptosis is the specific accumulation of lipid peroxides on the cell membrane, ultimately leading to cell membrane rupture and cell death. This process is initiated by the intracellular PUFAs. First, PUFAs are synthesized into PUFA-CoA under the action of Acyl-CoA synthetase long-chain family member 4 (ACSL4) [23]. Subsequently, LPCAT3 promotes the esterification of PUFA-CoA, incorporating it into phospholipids to generate phospholipids containing polyunsaturated fatty acids (PUFA-PLs) [24]. These PUFA-PLs can be oxidized by lipoxygenases (LOXs) or cytochrome P450 reductases (POR) under the catalysis of reactive oxygen species generated in the Fenton reaction, resulting in the formation of polyunsaturated fatty acid phospholipid hydroperoxides (PUFA-PLOOH). The excessive accumulation of PUFA-PLOOH disrupts the integrity of the cell membrane, ultimately triggering ferroptosis [25].

In contrast, monounsaturated fatty acids (MUFAs) such as oleic acid and palmitoleic acid, synthesized by Stearoyl-CoA desaturase 1 (SCD1), are less prone to lipid peroxidation due to their molecular structure, which lacks easily oxidizable double bonds. Therefore, MUFAs can competitively integrate into cell membrane phospholipids, replacing easily oxidizable PUFAs, thereby reducing the substrates for lipid peroxidation and exerting an inhibitory effect on ferroptosis [26–28].

### Imbalance of the antioxidant system

#### System Xc⁻-GSH-GPX4 Axis

The System Xc⁻-GSH-GPX4 axis is one of the core intracellular antioxidant pathways. This axis begins with the cystine-glutamate antiporter system (System Xc⁻), which is composed of the heterodimer formed by Solute carrier family 7 member 11 (SLC7A11) and Solute carrier family 3 member 2 (SLC3A2) [29]. Its function is to transport extracellular cystine into the cell while exporting glutamate. The cystine that enters the cell is reduced to cysteine, which is used for the synthesis of glutathione (GSH). Glutathione peroxidase 4 (GPX4) [30] is an antioxidant enzyme that inhibits lipid hydroperoxides by converting GSH to oxidized glutathione (GSSG) and transforming lipid hydroperoxides into phospholipids, thereby maintaining intracellular redox balance and inhibiting oxidative stress and ferroptosis [31].

#### FSP1-CoQ-NAD(P)H axis

The FSP1-CoQ-NAD(P)H axis is a powerful ferroptosis inhibition system that operates independently of GPX4 [32]. Its core mechanism involves the reduction of oxidized coenzyme Q_10_ (CoQ_10_) on the cell membrane to hydrogenated coenzyme Q10 (CoQ_10_H_2_), which possesses antioxidant activity. CoQ_10_H_2_, as a lipophilic antioxidant, can directly neutralize lipid radicals and lipid peroxides, interrupting the chain reaction of lipid peroxidation. Ferroptosis suppressor protein 1 (FSP1), also known as AIFM2, is the key enzyme in this pathway. In 2019, two independent research teams simultaneously discovered that FSP1 is primarily located in the cytoplasm and on the cell membrane, with its true key function being that of an NAD(P)H-CoQ_10_ reductase [32, 33]. It can utilize electrons provided by nicotinamide adenine dinucleotide (phosphate) hydride (NAD(P)H) to perform its NAD(P)H-CoQ_10_ reductase function [34]. The discovery of this pathway reveals the existence of a functionally redundant backup defense mechanism against ferroptosis in cells, explaining why the inhibition of GPX4 alone is sometimes limited in effectiveness [35].

#### DHODH-CoQH_10_ axis

The DHODH-CoQ_10_ axis is another ferroptosis defense system found to be independent of GPX4. This pathway is located in the inner mitochondrial membrane, utilizing the reducing power generated by dihydroorotate dehydrogenase (DHODH) in its natural enzymatic reaction (pyrimidine synthesis pathway) to reduce CoQ_10_ to CoQ_10_H_2_ within the mitochondria, thereby specifically protecting the mitochondrial membrane from lipid peroxidation. It works in conjunction with the FSP1-CoQ_10_ axis, which primarily acts on the cytoplasmic membrane, providing multi-layered spatial protection for the cell [36].

#### GCH1-BH4 axis

The GCH1/BH4 axis exerts an inhibitory effect on ferroptosis through tetrahydrobiopterin (BH4) [35]. Guanosine triphosphate (GTP) is synthesized into BH4 under the catalysis of GTP cyclohydrolase 1 (GCH1). BH4 serves as a direct reductant, reducing lipid peroxides to stable lipid alcohols on one hand [37]. On the other hand, BH4 acts as an important coenzyme, for instance, participating in the function of DHODH, thereby indirectly supporting the CoQ10 antioxidant system on the mitochondrial and cytoplasmic membranes, further enhancing the cellular resistance to ferroptosis [37, 38].

### Other ways

#### Nrf2

Nuclear factor E2-related factor 2 (Nrf2) is a fundamental transcription factor involved in cellular antioxidant stress responses and plays a pivotal role in the regulation of ferroptosis [39, 40].

Nrf2 suppresses ferroptosis by directly upregulating the expression of multiple key genes. On one hand, it increases the expression of the Glutamate-Cysteine ligase catalytic subunit (GCLC) and the modifying subunit (GCLM), thereby promoting the synthesis of GSH [41]. On the other hand, it directly activates the transcription of SLC7A11 and GPX4, comprehensively enhancing the antioxidant function of the System Xc⁻-GSH-GPX4 axis [40, 42].

Nrf2 also upregulates the expression of FTH1 and FTL [40], and activates heme oxygenase-1 (HO-1) [41]. HO-1 catalyzes the degradation of heme, resulting in the production of biliverdin, carbon monoxide (CO), and Fe^2^⁺ [43, 44]. Biliverdin can be further reduced to bilirubin, while both bilirubin and CO function as antioxidants that inhibit lipid peroxidation. However, the activation of HO-1 concurrently releases Fe^2^⁺, which may promote ferroptosis. Therefore, the overall effect of the Nrf2/HO-1 pathway on ferroptosis—whether it inhibits or promotes this process—depends on various factors, including the specific cellular context, the intensity of stress, and the temporal dynamics involved [44].

Kelch-Like ECH-Associated protein 1 (KEAP1) serves as a crucial negative regulator of Nrf2, typically suppressing its activity by binding to it and promoting its degradation [45, 46]. However, under conditions of oxidative stress, the autophagy adapter protein p62 is upregulated and competes with Nrf2 for binding to KEAP1. This competition stabilizes Nrf2, leading to its accumulation. Consequently, Nrf2 activates the transcription of downstream genes related to antioxidant responses and iron metabolism, ultimately enhancing the inhibition of ferroptosis [39, 41, 47].

#### p53

P53 is a crucial tumor suppressor protein, with distinct functions exhibited by its wild-type and mutant forms: the wild-type p53 induces tumor suppression, while the mutant variant may promote carcinogenesis [48]. Research indicates that wild-type p53 induces ferroptosis by directly suppressing the transcription of SLC7A11 [49], which reduces cysteine uptake and GSH synthesis. Additionally, p53 upregulates spermidine/spermine N1-Acetyltransferase 1 (SAT1), thereby promoting the production of hydrogen peroxide (H₂O₂) and directly exacerbating lipid peroxidation [50].

#### HSPB1

Heat shock protein family member B-1 (HSPB1), also known as Hsp27, serves as a critical intrinsic inhibitor of ferroptosis within cells. It exerts protective effects by maintaining redox homeostasis and stabilizing the cytoskeleton [51, 52]. HSPB1 stabilizes the actin cytoskeleton, and an intact cytoskeleton suppresses the endocytosis and recycling of TFR1, thereby reducing cellular iron uptake. Additionally, HSPB1 elevates intracellular GSH levels and enhances the activity of antioxidant enzymes, which collectively reduce the accumulation of ROS [53]. Furthermore, HSPB1 localizes to the mitochondria, where it helps maintain mitochondrial membrane integrity, mitigates mitochondrial ROS leakage, and protects mitochondria from ferroptosis-associated damage, such as reduced cristae and increased membrane density [54].

## Ferroptosis and the tumor microenvironment

The tumor microenvironment (TME) is a dynamic internal locale composed of tumor cells, immune cells (including T cells and macrophages), stromal cells, vascular endothelial cells, and non-cellular components such as the extracellular matrix, signaling molecules, and metabolic byproducts [55]. This microenvironment not only provides essential physical and biochemical support for tumor proliferation, invasion, and metastasis but also acts as the primary site for mediating immune evasion and therapeutic resistance [56, 57].

Tumor immunotherapy represents a significant focus in current clinical research, and tumor cell ferroptosis—a newly emerging form of programmed cell death—is being increasingly studied [58]. Given that the TME serves as a critical site for immunotherapy to exert its effects, investigating the occurrence of ferroptosis within the TME and its immune regulatory mechanisms has become a promising strategy for combination therapy [59].

Research has demonstrated that immune cells within TME can directly influence ferroptosis in tumor cells. For example, CD8 + T cells not only eliminate tumor cells through the perforin-granzyme pathway and the Fas/FasL pathway but also induce ferroptosis by secreting IFN-γ, which downregulates the expression of cystine transporter complexes, such as SLC3A2 and SLC7A11, in these cells [60]. Conversely, immune cells themselves exhibit varying sensitivities to ferroptosis, creating opportunities to reshape the immune microenvironment. Taking tumor-associated macrophages (TAMs) as an example, they are typically categorized into pro-inflammatory M1 macrophages, which possess anti-tumor activity, and pro-tumor, anti-inflammatory M2 macrophages. Studies indicate that M1 macrophages, which express high levels of inducible nitric oxide synthase (iNOS), display tolerance to ferroptosis. The nitric oxide (NO) they produce inhibits lipid peroxidation, thereby preventing ferroptosis. In contrast, pro-tumor M2 macrophages are relatively sensitive to ferroptosis [61]. Therefore, the use of ferroptosis inducers can selectively eliminate M2-type TAMs, reverse their immunosuppressive effects, and potentially promote macrophage polarization toward the M1 phenotype, yielding synergistic anti-tumor effects. For instance, in lung cancer, dihydroartemisinin induces TAM ferroptosis by activating the NF-κB signaling pathway, thereby facilitating their conversion to the M1 type [62]. The natural compound dictamnine can also trigger ferroptosis in tumor cells within colorectal cancer while simultaneously inhibiting M2 polarization of macrophages by blocking M2-associated markers such as CD163, TGF-β, and IL-10 [63]. This shift towards an M1-dominant anti-tumor state further confirms the role of ferroptosis in reshaping the cancer immune microenvironment.

In summary, the interaction between ferroptosis and the immune system within the tumor microenvironment constitutes a finely tuned regulatory network. On one hand, adaptive immunity, represented by CD8 + T cells, can directly attack tumors by inducing ferroptosis in tumor cells [60]. On the other hand, the differential sensitivity of innate immune cells, such as macrophages, to ferroptosis provides key targets for selectively reshaping the immune microenvironment through ferroptosis modulators [64]. This dual role of ferroptosis in the TME suggests that future therapeutic strategies may leverage a "bidirectional regulation" approach. First, a "pro-death" strategy aims to enhance the ability of immune cells to induce ferroptosis in tumor cells or directly employ ferroptosis inducers to eliminate tumors. Second, an "environment-shaping" strategy seeks to selectively eliminate immunosuppressive components, such as M2 macrophages, to potentially transform "cold tumors" into "hot tumors," thereby generating synergistic effects with immune checkpoint inhibitors (ICIs) [65, 66]. Importantly, as will be discussed in the following sections, many TCM-derived natural products exemplify this bidirectional logic: they not only directly trigger ferroptosis in cancer cells but also modulate the ferroptosis sensitivity and functional states of immune and stromal cells within the TME. Understanding this dual impact—on both tumor cells and their surrounding microenvironment—is essential for interpreting the mechanistic diversity of the compounds reviewed below.

## Natural products targeting ferroptosis against cancer

### Lung cancer

Lung cancer is one of the most prevalent malignant tumors and has the highest mortality rate among all cancers. Research indicates that lung cancer cells can enhance their antioxidant capacity by upregulating System Xc⁻, which suppresses ferroptosis [67]. Concurrently, FSP1 is highly expressed in lung cancer, providing protection to cells against ferroptosis-inducing agents and promoting the growth of lung cancer cells [33, 68].

**Fig. 2:**
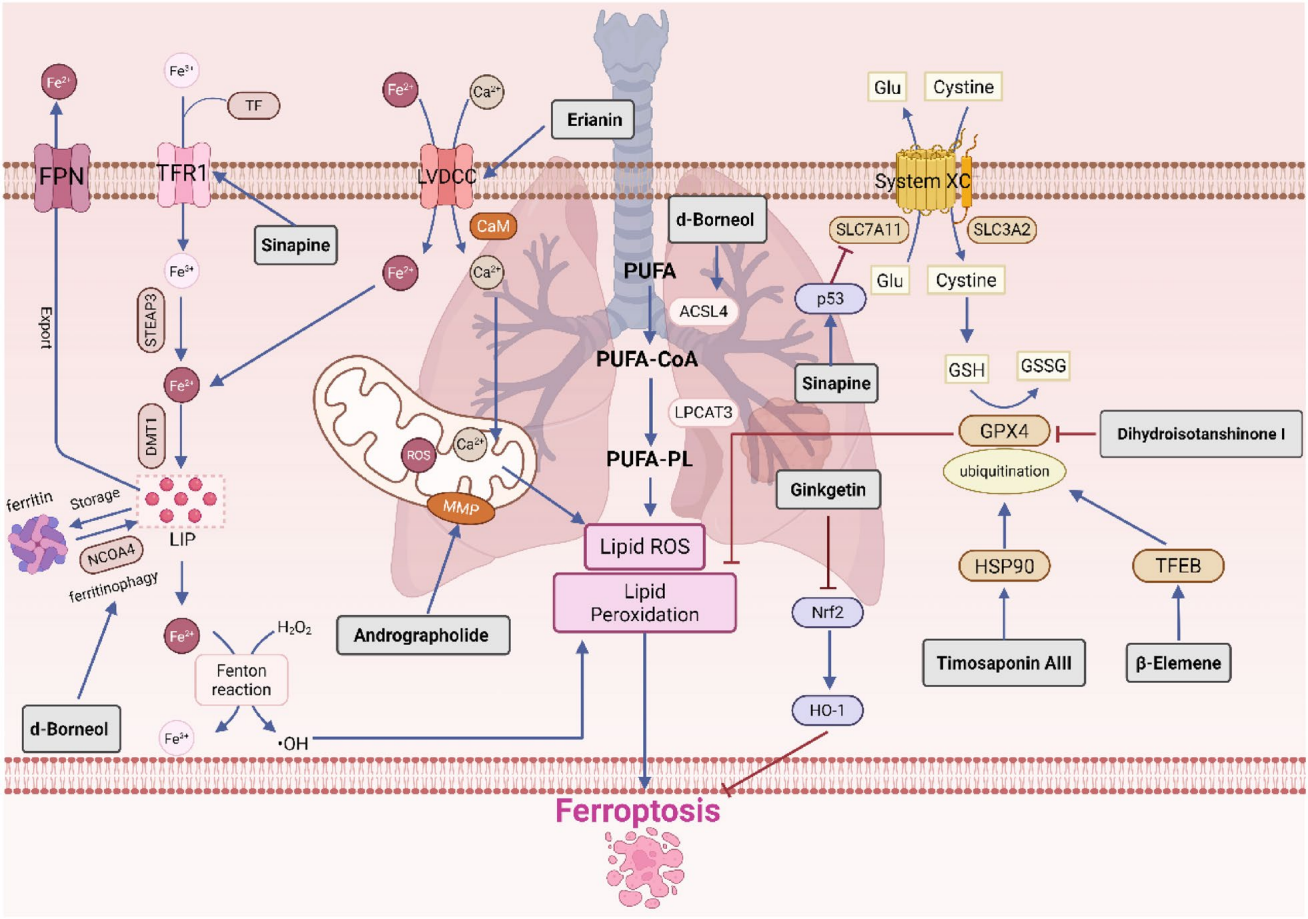
Natural products targeting ferroptosis against lung cancer

Timosaponin AIII is a steroid saponin derived from the rhizomes of Anemarrhena asphodeloides. Research indicates that Timosaponin AIII targets and enhances the HSP90-mediated ubiquitination and degradation of GPX4, thereby inducing ferroptosis in non-small cell lung cancer cells. This process is characterized by the release of reactive oxygen species, iron accumulation, malondialdehyde production, glutathione depletion, and a reduced mitochondrial membrane potential (MMP). Collectively, these events lead to cell cycle arrest, inhibition of proliferation and migration, and ultimately result in cell death [69]. Erianin is a dibenzyl compound extracted from Dendrobium plants. It activates the Ca^2^⁺/CaM signaling pathway and downregulates the expression of GSH, GPX4, CHAC2, SLC7A11, and glutaminase, while simultaneously upregulating levels of ROS, malondialdehyde (MDA), HO-1, and transferrin. This results in elevated intracellular Ca^2^⁺ and Fe^2^⁺ levels, inducing ferroptosis and inhibiting migration in lung cancer cells, thereby exerting anticancer activity [70]. Ginkgetin is the primary flavonoid component found in the leaves of Ginkgo biloba. Research has shown that ginkgo flavones can enhance the efficacy of cisplatin by modulating the Nrf2/HO-1 axis. This mechanism includes the downregulation of SLC7A11 and GPX4 expression, leading to a reduction in the GSH/GSSG ratio and a decrease in MMP. Further studies revealed that following combined treatment with Ginkgetin and cisplatin, the mRNA levels of SLC7A11 and GPX4 in cells exhibited no significant changes; however, their protein levels decreased markedly. Moreover, treatment with the lysosomal inhibitor chloroquine reversed the reduction in GPX4 protein, suggesting that the degradation of GPX4 may be mediated by the autophagy pathway [71]. Dihydroisotanshinone I is a diterpenoid quinone compound extracted from the roots of Salvia miltiorrhiza. This compound inhibits the growth of lung cancer cell lines A549 and H460 by downregulating GPX4 expression, which triggers lipid peroxidation and induces ferroptosis in the cancer cells [72].β-Elemene is a sesquiterpene compound derived from the rhizomes of Curcuma zedoaria. It binds to TFEB, significantly activating both TFEB and lysosomal function, while also upregulating the transcription of downstream genes such as GLA, MCOLN1, and SLC26A11. This process promotes the degradation of GPX4 through the lysosomal pathway, ultimately inducing ferroptosis in non-small cell lung cancer cells [73]. Andrographolide is a diterpenoid lactone derived from Andrographis paniculata. In non-small cell lung cancer cells, Andrographolide induces abnormal levels of ROS, GSH, and MDA, resulting in mitochondrial dysfunction characterized by increased mitochondrial ROS release, membrane potential depolarization, and reduced ATP synthesis [74]. Sinapine is an alkaloid extracted from cruciferous plants. Research has shown that sinapine enhances intracellular iron levels by upregulating transferrin and its receptors. Additionally, it induces ferroptosis in non-small cell lung cancer cells by inhibiting SLC7A11 expression in a manner dependent on p53 [75].d-Borneol is a bicyclic monoterpene compound. Research indicates that d-Borneol, in conjunction with cisplatin, induces ferroptosis via NCOA4-mediated ferritin autophagy. This mechanism enhances the expression of ACSL4, modulates PCBP2 and PRNP to facilitate the conversion of Fe^3^⁺ to Fe^2^⁺, reduces the activity and expression of antioxidant enzymes such as GSH and HO-1, and promotes the accumulation of ROS. Furthermore, the synergistic activation of autophagy inhibits the epithelial-mesenchymal transition process and increases the sensitivity of tumor cells to cisplatin [76] (Fig. 2).

### Breast cancer

According to 2022 global cancer research data, breast cancer ranks as the second most common cancer worldwide and is the fifth leading cause of cancer-related deaths [6]. It is the most frequently diagnosed cancer and the primary cause of cancer mortality among women. Recent studies indicate that breast cancer is characterized as an iron- and lipid-rich tumor, making the induction of ferroptosis a promising therapeutic strategy [77, 78].

**Fig. 3:**
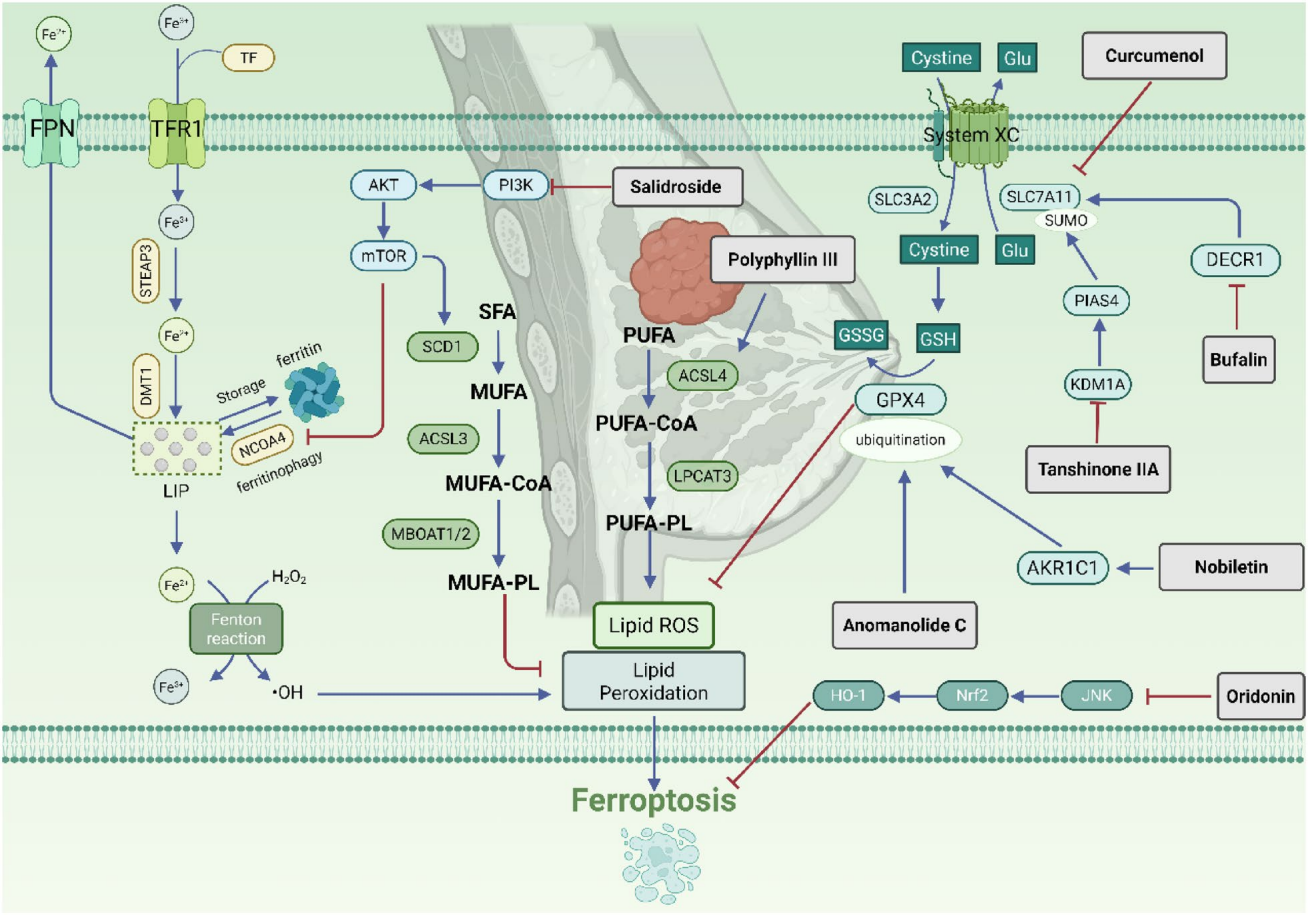
Natural products targeting ferroptosis against breast cancer

Salidroside, a glycoside compound extracted from Rhodiola rosea, has been shown to induce ferroptosis in triple-negative breast cancer cells by increasing intracellular Fe^2^⁺ levels. This effect occurs through the inhibition of the PI3K/AKT/mTOR signaling axis, suppression of SCD1-mediated monounsaturated fatty acid production and GPX4-mediated antioxidant defense, and promotion of NCOA4-mediated ferritin autophagy [79]. Tanshinone IIA, a terpenoid compound extracted from Salvia miltiorrhiza, downregulates KDM1A, which results in reduced transcriptional expression of PIAS4. The SUMOylation inhibitor 2-D08 enhances the ubiquitin-dependent degradation of SLC7A11, suggesting a competitive relationship between SUMOylation and ubiquitination for the lysine residues of SLC7A11, thereby protecting it from degradation. Consequently, Tanshinone IIA promotes ferroptosis in breast cancer cells by inhibiting PIAS4-mediated SUMOylation of SLC7A11, leading to the instability and degradation of SLC7A11 protein [80]. Anomanolide C, a withanolide compound isolated from Tubocapsicum anomalum, has been found to reduce GPX4 protein levels by inducing its ubiquitination and degradation, resulting in Fe^2^⁺ accumulation and subsequently inducing autophagy-dependent ferroptosis in triple-negative breast cancer [81]. Nobiletin, a polymethoxyflavone derived from citrus peel, promotes ferroptosis in breast cancer cells by targeting and enhancing AKR1C1-mediated ubiquitination and degradation of GPX4, accompanied by the release of ROS, iron accumulation, MDA production, and depletion of GSH [82]. Oridonin, a tetracyclic diterpene compound derived from wintergreen, has been shown to enhance the ferroptosis effect induced by the ferroptosis inducer RSL3, which operates through the inhibition of GPX4 activity. This enhancement is mediated by the modulation of the JNK/Nrf2/HO-1 oxidative stress signaling pathway [83]. Curcumenol is a sesquiterpene compound derived from plants in the ginger family, such as Curcuma zedoaria. Research indicates that curcumenol induces ferroptosis in triple-negative breast cancer cells by inhibiting the SLC7A11/NF-κB/TGF-β signaling pathway [84]. Polyphyllin III, a steroidal saponin derived from Paris polyphylla, has been shown to upregulate ACSL4 simultaneously at both the transcriptional and protein levels, increase ROS levels, and concurrently decrease GPX4 and GSH levels. These effects collectively inhibit the proliferation of MDA-MB-231 cells by inducing ferroptosis [85]. Bufalin is a potent cardiac steroidal compound derived from toad venom. DECR1, a coenzyme involved in fatty acid β-oxidation, is highly expressed in breast cancer and plays a significant role in tumor progression. Research has shown that bufalin and its derivatives effectively suppress DECR1 expression and promote its degradation through autophagy and ubiquitination pathways. Furthermore, bufalin modulates levels of MDA, TG, ROS, and Fe^2^⁺ while downregulating the expression of HSL, FPN, SLC7A11, and GPX4, thereby inducing ferroptosis in MDA-MB-231 cells. Subsequent mechanistic studies revealed an interaction between SLC7A11 and DECR1. Inhibition of SLC7A11 resulted in decreased DECR1 expression and changes in ferroptosis-related markers, an effect that could be reversed by overexpressing DECR1. Collectively, these findings demonstrate that bufadienolide induces ferroptosis in breast cancer cells by modulating the DECR1-SLC7A11 axis [86] (Fig. 3).

### Colorectal cancer

Colorectal cancer (CRC) ranks as the third most common malignant tumor globally. Due to a widespread lack of awareness regarding early cancer screening, most patients are diagnosed at an advanced stage. Despite the numerous treatment options currently available for CRC, therapeutic outcomes for patients with advanced disease remain suboptimal. Recent studies indicate a close association between the occurrence and progression of CRC and ferroptosis. Given that CRC is frequently accompanied by characteristic abnormalities in iron metabolism and imbalances in oxidative stress, targeting the ferroptosis pathway has emerged as a highly promising anti-tumor strategy [87–89].

**Fig. 4:**
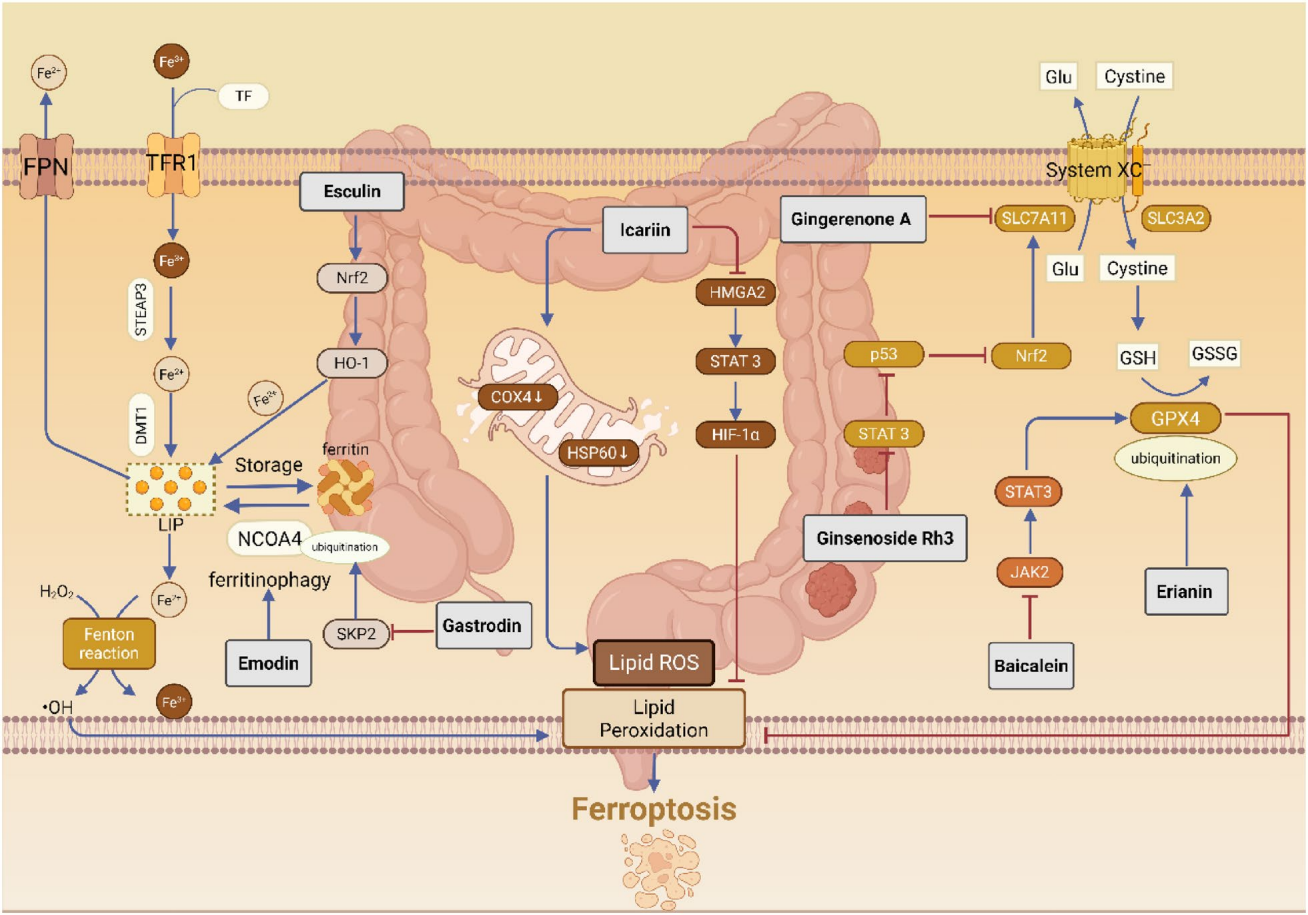
Natural products targeting ferroptosis against colorectal cancer

Erianin induces ferroptosis in colorectal cancer cells by promoting the ubiquitination and degradation of GPX4, which leads to increased intracellular levels of Fe^2^⁺ and MDA, while decreasing the GSH/GSSG ratio [90]. Furthermore, additional studies indicate that erianin also suppresses the growth and metastasis of KRASG13D-mutated colorectal cancer via an autophagy-dependent ferroptosis pathway [91].Baicalein, the primary flavonoid compound found in dried roots of Scutellaria baicalensis, has been shown to induce ferroptosis by targeting and inhibiting the JAK2/STAT3/GPX4 signaling pathway. The mechanism is as follows: Baicalein directly inhibits JAK2 kinase activity, which in turn reduces the phosphorylation of STAT3. This reduction weakens STAT3's transcriptional activation of the GPX4 gene, leading to a significant downregulation of both GPX4 mRNA and protein expression. Consequently, the decrease in GPX4 hampers the cell's ability to eliminate lipid peroxides, resulting in their accumulation and ultimately inducing ferroptosis in colorectal cancer cells [92].Emodin is a naturally occurring anthraquinone compound that is widely distributed in various plant species. It serves as the primary active ingredient in several laxative Chinese herbal medicines and exhibits broad-spectrum antitumor activity. Research indicates that emodin reduces GSH levels, downregulates the expression of xCT and GPX4, and increases the production of ROS, MDA content, and lipid peroxidation levels. Notably, ferroptosis inhibitors such as ferrostatin-1, iron chelators like deferoxamine (DFO), autophagy inhibitors such as 3-methyladenine (3-MA), and NCOA4 knockdown can reverse these effects. Furthermore, emodin inhibits the NF-κB pathway, while its activator phorbol 12-myristate 13-acetate (PMA) mitigates emodin-induced ferroptosis. Collectively, these findings suggest that emodin induces ferroptosis in colorectal cancer cells via NCOA4-mediated mechanisms through the inhibition of the NF-κB pathway [93].Gingerenone A is a phenolic compound isolated from ginger. Research has demonstrated that Gingerenone A directly interacts with SLC7A11, inducing its ubiquitination and subsequent degradation, which reduces the stability of the SLC7A11 protein. This process results in the depletion of GSH, accompanied by a significant accumulation of MDA, ROS, and 4-hydroxynonenal (4-HNE), ultimately leading to ferroptosis in colorectal cancer cells [94].Icariin, the principal flavanol glycoside derived from Epimedium plants, has been shown to promote ferroptosis in colorectal cancer cells by inducing mitochondrial dysfunction and inhibiting the HMGA2/STAT3/HIF-1αsignaling pathway. Furthermore, it synergistically enhances the efficacy of PD-1 inhibitors. The mechanism underlying this action involves the induction of mitochondrial dysfunction, which results in elevated levels of mitochondrial ROS through the Fenton reaction, a process driven by the accumulation of free iron. This sequence of events promotes lipid peroxidation, ultimately leading to ferroptosis and effectively inhibiting tumor growth [95].Esculin is the primary coumarin glycoside component of the TCM herb Phellodendron amurense. It induces endoplasmic reticulum stress in colorectal cancer cells by regulating the PERK-mediated eIF2α/CHOP and Nrf2/HO-1 cascades, thereby promoting apoptosis and ferroptosis. Specifically, esculin activates PERK, which leads to the phosphorylation of eIF2α. This phosphorylation upregulates CHOP expression, triggering apoptosis associated with ER stress. Concurrently, esculin suppresses the Nrf2/HO-1 pathway, which reduces cellular antioxidant capacity. Consequently, this results in elevated levels of ROS and exacerbates lipid peroxidation, ultimately inducing ferroptosis [96].Ginsenoside Rh3 is a rare protoginsenoside that has been shown to induce pyroptosis and ferroptosis in colorectal cancer cells through the Stat3/p53/Nrf2 signaling axis. Specifically, Rh3 inhibits the nuclear translocation of Nrf2 and reduces the expression of HO-1, which in turn promotes the expression of NLRP3 and caspase-1, ultimately leading to the activation of GSDMD-dependent pyroptosis. Additionally, Rh3 suppresses the expression of SLC7A11 by preventing Nrf2 from entering the nucleus, resulting in GSH depletion, iron accumulation, increased ROS production, and MDA accumulation, thereby inducing ferroptosis [97].Gastrodin is a phenolic glycoside compound derived from the dried rhizomes of Gastrodia elata. In CRC cells, treatment with gastrodin inhibits the expression of SKP2 protein. SKP2, a pivotal component of the SCF ubiquitin ligase complex, facilitates the ubiquitin-mediated degradation of NCOA4. A reduction in SKP2 levels results in the inhibition of NCOA4 degradation, leading to an increase in NCOA4 expression. As a significant mediator of ferroptosis, the elevated expression of NCOA4 promotes the degradation of ferritin, which causes intracellular iron overload and ultimately induces ferroptosis [98] (Fig. 4).

### Gastric cancer

Gastric cancer (GC) is a malignant tumor characterized by a high risk of recurrence and has emerged as one of the leading causes of cancer-related deaths globally. This phenomenon is particularly pronounced in East Asian countries, such as China, Japan, and South Korea, where the incidence rates of GC are significantly higher than those observed in Western nations [99]. Research has demonstrated a close association between iron levels and gastric health. Anemia, low ferritin levels, and iron malabsorption linked to autoimmune gastritis are all recognized as risk factors for gastrointestinal tumors and gastric cancer [100–102]. Consequently, iron deficiency plays a critical role in the occurrence, progression, treatment, and prognosis of GC [103].

**Fig. 5:**
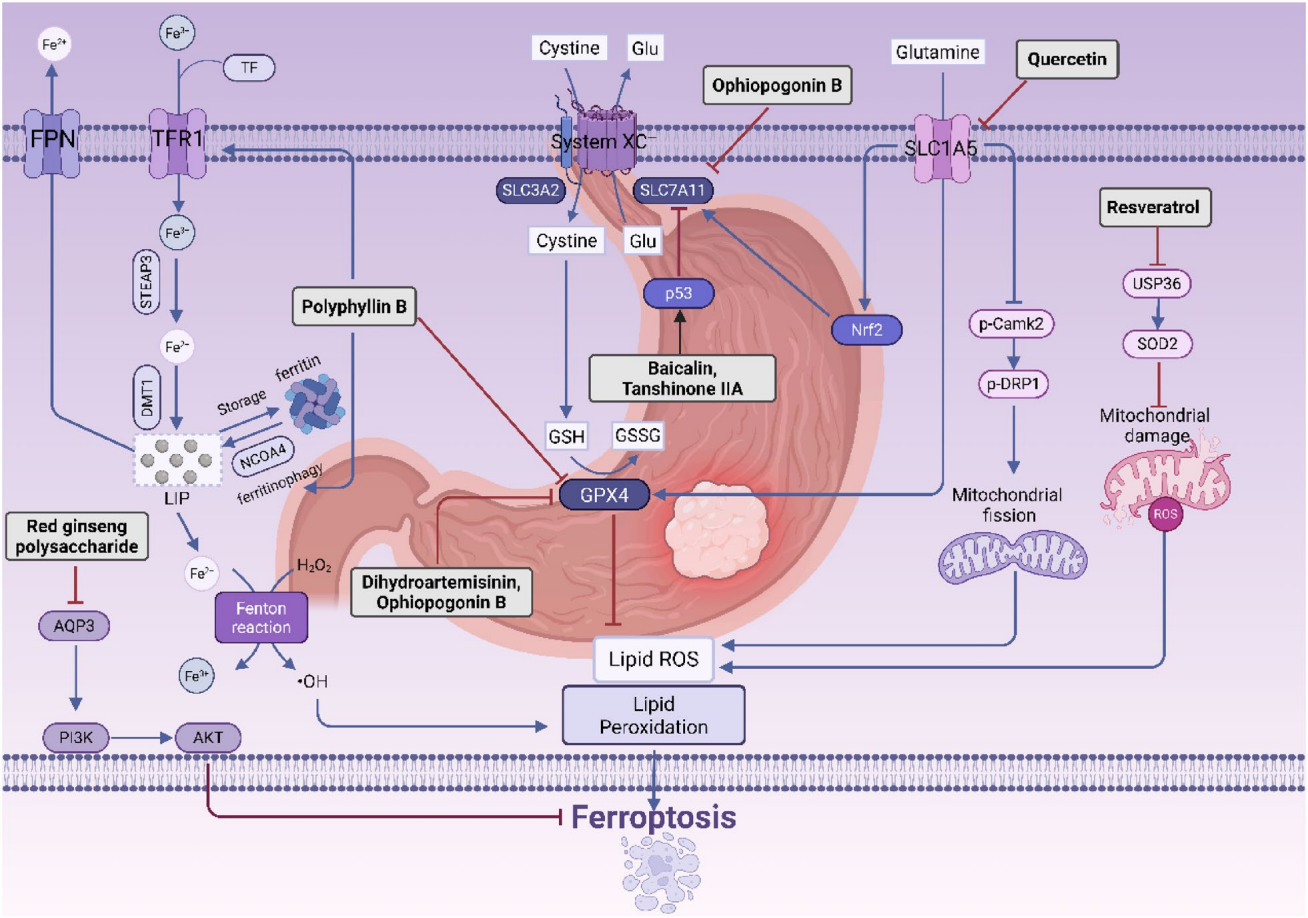
Natural products targeting ferroptosis against Gastric cancer

Polyphyllin B is a steroidal saponin derived from the dried rhizomes of Paris polyphylla or Paris polyphylla var. japonicus, both of which belong to the Liliaceae family. Research indicates that Polyphyllin B, recognized as a novel GPX4 inhibitor, can modulate the expression of LC3B, TFR1, NCOA4, and FTH1 in in vitro studies. This suggests that Polyphyllin B may facilitate the transport of Fe^3^⁺ into cells via TFR1, promote NCOA4-dependent ferroptosis, thereby increasing intracellular Fe^2^⁺ levels and downregulating GPX4 expression in gastric cancer cells, ultimately leading to the induction of ferroptosis [104]. Quercetin is a flavonoid compound that is widely present in various plants and exhibits multiple physiological activities, including antioxidant, anti-inflammatory, and anti-allergic effects. In gastric cancer cells, quercetin inhibits Nrf2 by targeting SLC1A5, which leads to a reduction in xCT/GPX4 expression and the activation of the p-Camk2/p-DRP1 pathway. This activation subsequently upregulates p-DRP1, resulting in increased mitochondrial fission and the release of reactive oxygen species (ROS). Furthermore, quercetin enhances intracellular iron accumulation and accelerates iron deposition by inhibiting SLC1A5. Collectively, these effects promote ferroptosis in gastric cancer cells, thereby inhibiting tumor progression [105]. Baicalin, the primary flavonoid component in dried Scutellaria baicalensis root, exerts antitumor effects in gastric cancer through multiple pathways and targets. It promotes reactive oxygen species-mediated ferroptosis and enhances the efficacy of 5-fluorouracil (5-FU). When combined with 5-FU, baicalin upregulates the expression of TFR1, NOX1, and COX2 while downregulating FTH1, FTL, and GPX4 [106]. Additionally, baicalin disrupts iron homeostasis and suppresses antioxidant defenses, leading to iron accumulation and lipid peroxide aggregation. Specifically, it upregulates p53 expression, inhibits the p53-mediated SLC7A11/GPX4 pathway, and increases ROS accumulation, thereby activating ferroptosis, suppressing the viability of oxaliplatin-resistant HGC27/L cells, and enhancing their sensitivity to oxaliplatin-based chemotherapy [107]. Resveratrol is a natural polyphenol present in various plants, including grapes, peanuts, and Polygonum cuspidatum. USP36 stabilizes SOD2 through deubiquitination, thereby preserving mitochondrial integrity and facilitating tumor progression. However, resveratrol disrupts the USP36-SOD2 axis, leading to a reduction in SOD2 stability and the induction of mitochondrial dysfunction. This process triggers autophagy and ferroptosis in gastric cancer cells [108]. Dihydroartemisinin is the principal active metabolite of artemisinin. Research has demonstrated that the combination of Dihydroartemisinin and cisplatin synergistically inhibits the proliferation, invasion, and migration of gastric cancer cells. This effect is mediated by the suppression of GPX4 and the induction of ferroptosis [109]. Red ginseng polysaccharide is a water-soluble active component derived from red ginseng. Aquaporin 3 (AQP3) is a transmembrane aquaporin that is highly expressed in gastric cancer and is associated with tumor proliferation and anti-apoptotic mechanisms. Research indicates that Red ginseng polysaccharide inhibits the PI3K/Akt signaling pathway by downregulating AQP3 expression, thereby promoting ferroptosis in AGS cells. This process reduces the proliferation capacity and viability of these cells, ultimately leading to cell death [110]. Tanshinone IIA promotes lipid peroxidation and upregulates the expression of ferroptosis-related genes, such as Ptgs2 and Chac1, while simultaneously reducing levels of GSH and Cys and increasing ROS. Studies indicate that the knockout of the p53 gene mitigates Tanshinone IIA-induced lipid peroxidation, suggesting that p53 plays a role in this ferroptotic process [111]. Ophiopogonin B is a steroidal saponin derived from the rhizomes of Ophiopogon japonicus. Research has shown that Ophiopogonin B significantly increases the mRNA levels of ferroptosis-associated markers Ptgs2 and Chac1 at the transcriptional level. It induces cell death in gastric cancer by inhibiting the GPX4/xCT-dependent ferroptosis pathway, an effect that can be reversed by pretreatment with Fer-1 [112] (Fig. 5).

### Liver cancer

Hepatocellular carcinoma (HCC) ranks as the sixth most common cancer worldwide and is the third leading cause of cancer-related mortality. Research has established a close association between ferroptosis and resistance to the targeted drug sorafenib in hepatocellular carcinoma. Additionally, ferroptosis inducers have been shown to exert antitumor effects and can synergize with sorafenib [113, 114]. Therefore, a comprehensive investigation into the mechanisms underlying ferroptosis in the progression of hepatocellular carcinoma may uncover novel pathogenesis and potential therapeutic targets, ultimately enhancing patient prognosis [115].

**Fig. 6:**
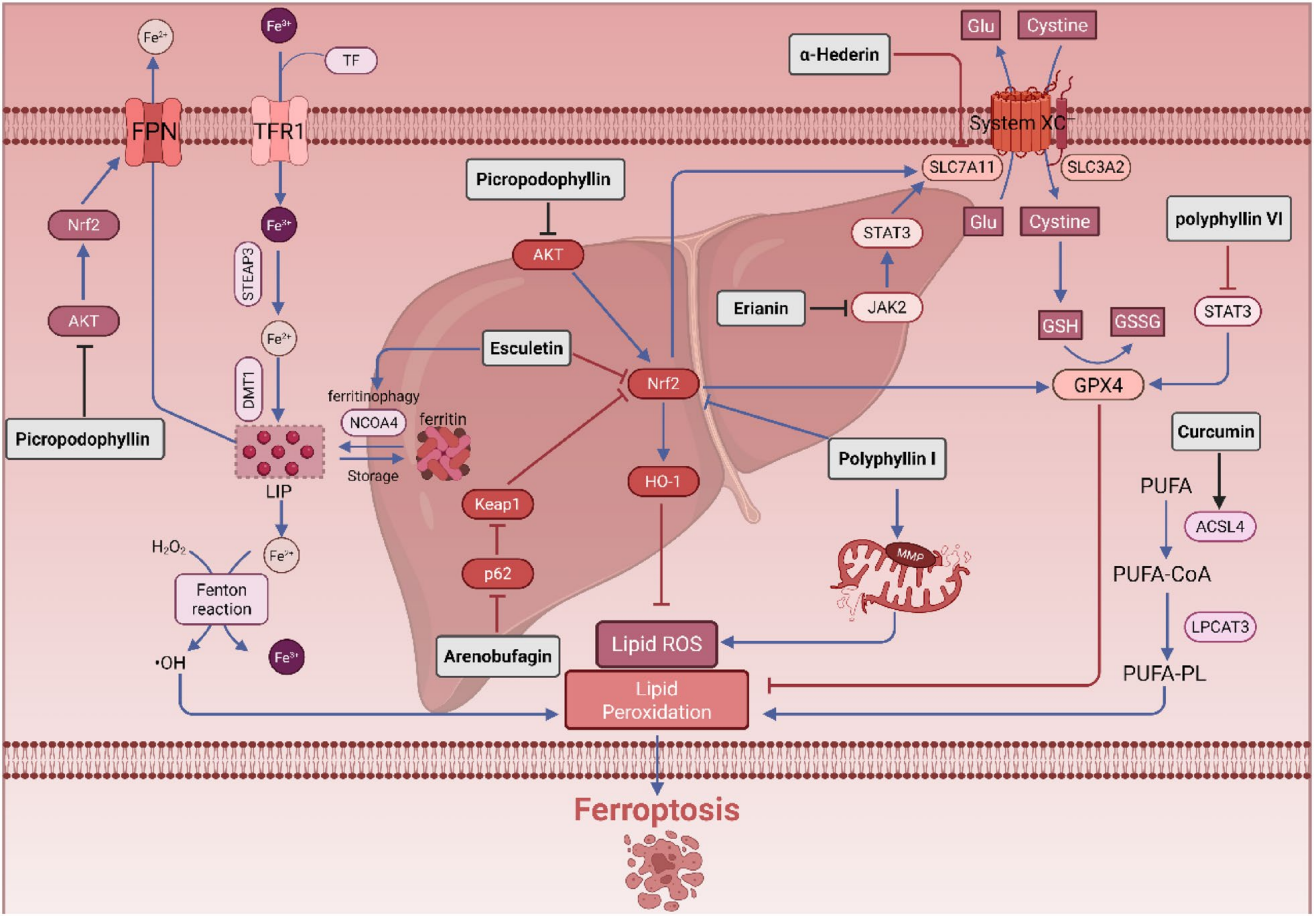
Natural products targeting ferroptosis against liver cancer

Polyphyllin I and Polyphyllin VI are steroidal saponins derived from the dried rhizomes of Paris polyphylla. Current research indicates that Polyphyllin I can induce ferroptosis by influencing the Nrf2/HO-1/GPX4 antioxidant axis through its binding and regulation of Nrf2, HO-1, and GPX4 proteins. Concurrently, Polyphyllin I induces structural damage to mitochondria and reduces membrane potential, leading to increased levels of ROS and MDA, enhanced accumulation of Fe^2^⁺, depletion of GSH, and suppression of xCT and GPX4 expression. Ultimately, Polyphyllin I exerts a dose-dependent inhibitory effect on the proliferation, invasion, and metastasis of HCC cells [116]. Network pharmacology and molecular docking analyses have revealed that Polyphyllin VI may target STAT3. Further studies have confirmed that STAT3 binds to GPX4 and promotes its expression. Therefore, Polyphyllin VI induces ferroptosis by inhibiting the STAT3/GPX4 axis, which subsequently suppresses HCC cell invasion and metastasis [117]. Esculetin, a natural coumarin derivative isolated from Eucommia ulmoides, has been shown to induce Fe^2^⁺ accumulation, disrupt mitochondrial morphology, elevate ROS and lipid peroxidation levels, and reduce glutathione peroxidase activity in hepatocellular carcinoma cells. This effect can be reversed by the ferroptosis inhibitor Fer-1 or by overexpression of Nrf2, confirming that esculetin exerts its anti-hepatocellular carcinoma activity by inhibiting ferroptosis through the Nrf2-xCT/GPX4 axis [118]. Furthermore, studies have demonstrated that Esculetin upregulates the expression of NCOA4, LC3-II, and lysosomal marker proteins in HUH7 and HCCLM3 cells, while simultaneously decreasing FTH1 protein levels. This indicates that Esculetin also promotes ferroptosis via the NCOA4-mediated ferritin autophagy pathway [119]. Arenobufagin is a potent cardiac steroidal compound derived from toad venom. It promotes autophagy-dependent ferroptosis in HepG2 cells by inducing autophagy and modulating the p62-Keap1-Nrf2 pathway. Additionally, it inhibits the expression of ferroptosis-related proteins Nrf2 and COX-2 in a dose-dependent manner, reduces levels of GSH and total SOD, and increases MDA levels [120]. Picropodophyllin is a cyclolipid natural product derived from the root of the plant Dysosma versipellis in the Berberidaceae family, known for its anticancer properties against various solid tumors. As a natural inhibitor of IGF1R, Picropodophyllin induces ferroptosis in hepatocellular carcinoma by obstructing the AKT/Nrf2/SLC7A11 and AKT/Nrf2/SLC40A1 signaling pathways [121]. Curcumin is a natural polyphenol derived from the rhizomes of turmeric. Following the intervention with curcumin, there was a significant increase in intracellular levels of MDA and Fe^2^⁺, while levels of GSH decreased. Additionally, the expression of GPX4 and SLC7A11 proteins was downregulated, whereas the expression of ACSL4 and PTGS2 was upregulated. These findings suggest that curcumin promotes ferroptosis in hepatocellular carcinoma cells through the upregulation of ACSL4 [122].α-Hederin is a triterpene saponin extracted from the yellow–brown honeysuckle. Research indicates that α-Hederin suppresses the expression of xCT in hepatocellular carcinoma cells, downregulates levels of GSH and GPX4, and simultaneously upregulates the expression of DMT1. This process promotes the transport of Fe^2^⁺ to the unstable iron pool, thereby disrupting intracellular iron metabolism. Through the Fenton reaction, α-Hederin generates substantial lipid ROS, ultimately inducing ferroptosis [123]. Erianin promotes ferroptosis and inhibits invasion in HCC through the JAK2/STAT3/SLC7A11 pathway, thereby suppressing tumor growth. It achieves this by blocking STAT3 activation through the inhibition of JAK2 phosphorylation, which in turn reduces STAT3's upregulation of SLC7A11. Consequently, this process leads to increased levels of intracellular lipid peroxidation, total iron content, Fe^2^⁺ levels, and ROS, ultimately inducing ferroptosis in HCC cells [124] (Fig. 6).

## Discussion

**Fig. 7:**
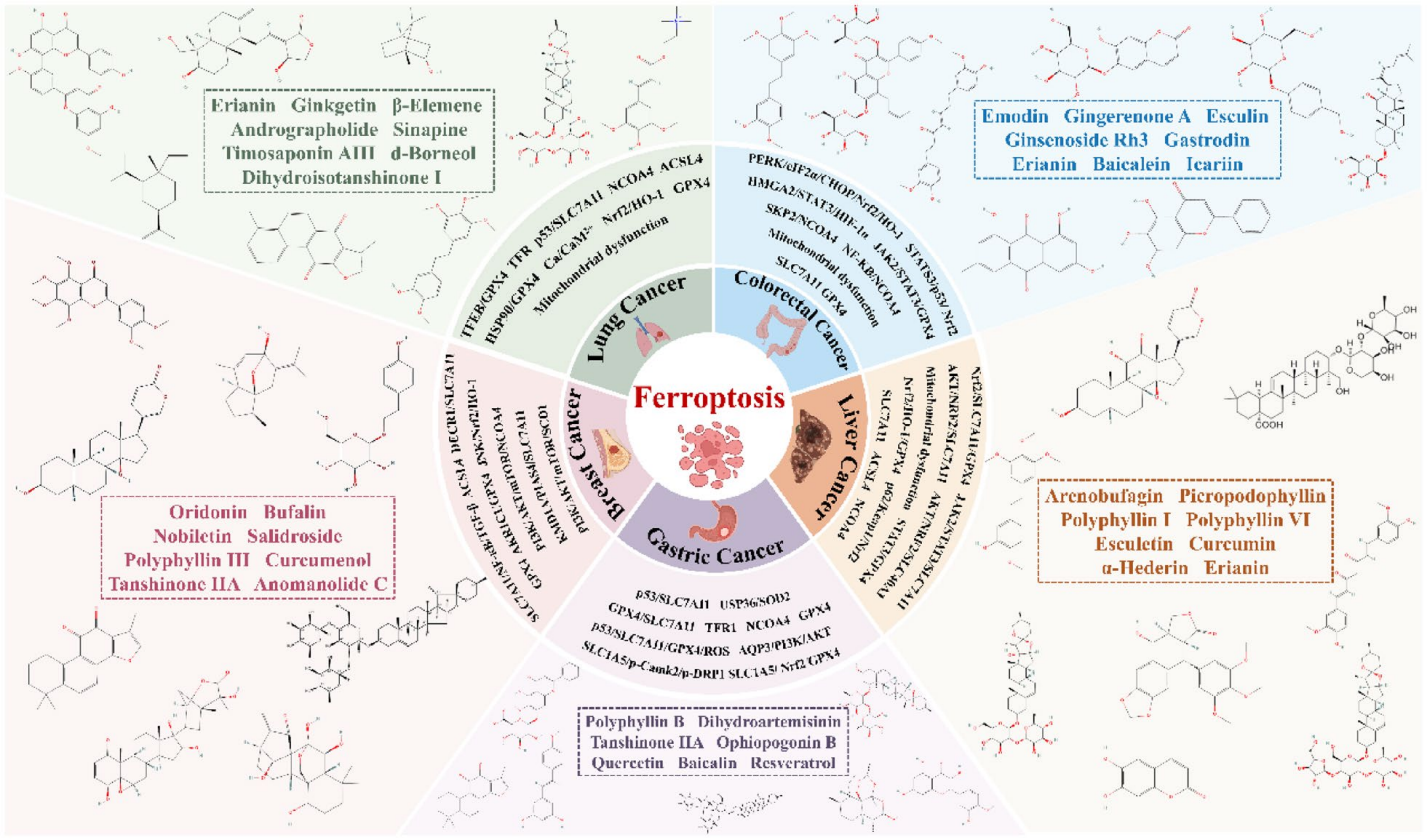
Natural products modulate ferroptosis mechanisms in different cancers

Ferroptosis, a form of programmed cell death driven by iron-dependent lipid peroxidation, presents novel therapeutic targets for cancer treatment due to its unique regulatory mechanisms. This review systematically summarizes current research on TCM natural products that combat lung, breast, colorectal, gastric, and liver cancers through the modulation of ferroptosis. It elucidates the multidimensional effects of these natural products on the ferroptosis pathway, thereby providing a reference for the development of TCM-based anticancer drugs (Table 1 and Fig. 7).

**Table 1:** Nature products targeting ferroptosis against cancer

Component	Structure	Classification	Cancer type	Mechanisms	In vivo model (Dose)	In vitro model (Concentration)	Refs.
Timosaponin AIII		Steroids	NSCLC	Facilitating HSP90 mediated GPX4 ubiquitination and degradation	C57BL/6 J and BALB/c nude mice (12.5 mg/kg and 50 mg/kg)	H1299, A549, SPC-A1, LLC (4 μM)	[69]
Erianin		Phenols	NSCLC	Activating Ca2 + /CaM signaling pathway	BALB/c nude mice (100 mg/kg)	H460 and H1299(12.5, 25, 50, 100 nM)	[70]
			CRC	Facilitating the ubiquitination and degradation of GPX4	C57BL/6 mice (20, 40, 80 mg/kg)	HCT116 and SW480 cells (25, 50, 100 nM)	[90]
			KRAS^G13D^ CRC	Interacting with KRAS^G13D^ and inducing autophagy-dependent ferroptosis	BALB/c nude mice (100 mg/kg)	LoVo and HCT116 Cells (25, 50, 100 nM)	[91]
			HCC	Blocking the JAK2/STAT3/SLC7A11 signaling pathway	BALB/c nude mice (20 mg/kg)	Huh7 and HepG2 cells (40, 80 nM)	[124]
Ginkgetin		Flavonoids	NSCLC	Inhibiting Nrf2/HO-1 signaling pathway	Xenograft nude mice (30 mg/kg)	A549 (5 μM)	[71]
Dihydroisotanshinone I		Terpenoids	Lung cancer	Blocking the protein expression of GPX4	Xenograft nude mice (30 mg/kg)	A549, H460, IMR-90 (20–30 μM)	[72]
β-Elemene		Terpenoids	NSCLC	Activating TFEB-mediated GPX4 degradation	NOD/SCID (120 mg/kg)	A549, NCI-H460, SPC-A-1 cells (120 μg/mL)	[73]
Andrographolide		Terpenoids	NSCLC	Activating mitochondrial dysfunction	C57BL/6 mice (5 mg/kg and 10 mg/kg)	H460 and H1650 cells (10–30 μM)	[74]
Sinapine		Alkaloids	NSCLC	Upregulating p53, TF, TFRC; downregulating SLC7A11	BALB/c mice (10 mg/kg, 20 mg/kg, 40 mg/kg)	A549, SK, H66, H460 and HBE cells (20 μM)	[75]
d-Borneol		Terpenoids	NSCLC	Promoting NCOA4-mediated ferritinophagy	BALB/c nude mice (30 mg/kg and 60 mg/kg)	H460/CDDP cells (2 μg/mL)	[76]
Salidroside		Glycosides	TNBC	Suppressing SCD1-mediated lipogenesis of MUFA and GPX4-mediated antioxidant defense; facilitating NCOA4-mediated ferritinophagy	BALB/c nude mice (100 mg/kg)	MDA-MB-231 cells (50 µM)	[79]
Tanshinone IIA		Terpenoids	Breast cancer	Suppressing KDM1A/PIAS4/SLC7A11 axis	BALB/c nude mice (10 mg/kg)	MCF-7, T47D, MDA-MB-231, BT549 cells (25, 50, 100, 200 µM)	[80]
			GC	Inducing SLC7A11 downregulation by p53 upregulation	NOD-SCID mice (50 mg/kg)	BGC-823 and NCI-H87 cells (2, 4 µM)	[111]
Anomanolide C		Esters	TNBC	Ubiquitinating GPX4-driven autophagy	BALB/c nude mice (25 mg/kg and 50 mg/kg)	MCF-10A, MDA-MB-231 and BT549 cells (0.25, 0.5, 1, 2 µM)	[81]
Nobiletin		Flavonoids	TNBC	Activating AKR1C1-mediated ubiquitination and degradation of GPX4	BALB/c mice (30 mg/kg and 60 mg/kg)	MDA-MB-231 and 4T1 cells (10, 20, 40 µM)	[82]
Oridonin		Terpenoids	Breast cancer	Activating the JNK/Nrf2/HO-1 axis	–	MCF-7 and MDA-MB-231 cells (10, 15 µM)	[83]
Curcumenol		Terpenoids	TNBC	Suppressing the SLC7A11/NF‑κB/TGF‑β signaling pathway	BALB/c mice (5 and 10 mg/kg)	4T1 and MDA‑MB‑231 cells (25, 50, 100 µM)	[84]
Polyphyllin III		Steroids	TNBC	Up-regulating ACSL4	BALB/C nude mice (5 mg/kg)	MDA‑MB‑231 (5 µM)	[85]
Bufalin		Steroids	Breast cancer	Suppressing the DECR1-SLC7A11 axis	BALB/c mice (1 mg/kg)	MDA-MB-231 (0.1, 0.2, 0.4 µM)	[86]
Baicalein		Flavonoids	CRC	Blocking the JAK2/STAT3/GPX4 axis	BALB/c nude mice (10 and 20 mg/kg)	HCT116 and DLD1 (7.5, 15, 30 µM)	[92]
Emodin		Anthraquinones	CRC	Promoting NCOA4-mediated ferritinophagy and suppressing the NF-κb pathway	BALB/c nude mice (10 and 20 mg/kg)	HCT-15 and SW620 (40, 80, 120 µM)	[93]
Gingerenone A		Phenols	CRC	Suppression of SLC7A11 signaling pathway	BALB/c nude mice (5, 10 and 20 mg/kg)	HCT-15 and HCT-116 cells (10, 20, 40, 80 µM)	[94]
Icariin		Flavonoids	CRC	Activating mitochondrial dysfunction	C57BL/6 mice (10, 20, 40 mg/kg)	HCT116, SW620, MC38, CT26 (40, 80 µM)	[95]
Esculin		Coumarins	CRC	Activating the PERK-eIF2α-CHOP and PERK-Nrf2-HO-1 signaling pathways	C57BL/6 mice (20 and 40 mg/kg)	HCT116 (40, 80 µM)	[96]
Ginsenoside Rh3		Saponins	CRC	Regulating STAT3/p53/ Nrf2 axis	BALB/c nude mice (20 mg/kg)	HT29 and HCT116 (20, 40, 80 µM)	[97]
Gastrodin		Glycosides	CRC	Inhibiting SKP2 to reduce NCOA4 ubiquitination	–	HCT116 (20 µM)	[98]
Polyphyllin B		Steroids	GC	Reducing the expression of GPx4, promoting iron ion transport and ferritinophagy	BALB/c nude mice (2.5 mg/kg and 5.0 mg/kg)	MKN-1 and NUGC-3 cells (0.5, 1, 2 µM)	[104]
Quercetin		Flavonoids	GC	Targeting SLC1A5, inhibiting the Nrf2/xCT pathway, activating the p-Camk2/p-DRP1 pathway, and accelerating iron deposition	BALB/c nude mice (20 mg/kg)	AGS and SNU-1 cells (40 µM)	[105]
Baicalin		Flavonoids	GC	Promoting ROS-mediated ferroptosis	–	AGS and SGC-7901(60 ng/mL and 90 ng/mL)	[106]
				Activating p53‑mediated SLC7A11/GPX4/ROS pathway	–	HGC-27 and HGC27/L cells (200, 250, 300 µM)	[107]
Resveratrol		Polyphenols	GC	Inhibiting USP36-mediated stabilization of SOD2 to induce mitochondrial damage and ROS accumulation	BALB/c nude mice (100 mg/kg)	SNU-5 and AGS cells (37.5 µM)	[108]
Dihydroartemisinin		Terpenoids	GC	Inhibiting GPX4	BALB/c nude mice (50 mg/kg)	SGC-7901 and HGC-27 (25 µM)	[109]
Red ginseng polysaccharide	-	Polysaccharides	GC	Inhibiting PI3K/Akt pathway through down-regulation of AQP3	BALB/c nude mice (75, 150, 300 mg/kg)	RGM-1 and AGS cells (50, 100, 200 µM)	[110]
Ophiopogonin B		Steroids	GC	Blocking the GPX4/xCT	Nude mice (50 mg/kg)	AGS and NCI‑N87 cells (10, 20 µM)	[112]
Polyphyllin I		Steroids	HCC	Activating the mitochondrial dysfunction via Nrf2/HO-1/GPX4 axis	BALB/c nude mice (1.5 mg/kg and 3 mg/kg)	HepG2 and MHCC97H cells (2, 4, 6 µM)	[116]
Polyphyllin VI		Steroids	HCC	Inhibiting the STAT3/GPX4 axis	BALB/c nude (10 mg/kg)	HCCLM3 and Huh7 Cells (2, 4, 6 µM)	[117]
Esculetin		Coumarins	HCC	Inhibiting the Nrf2-xCT/GPx4 axis	Zebrafish (280 µM)	Hep3B cells (28, 56, 112 µM)	[118]
				Promoting NCOA4 pathway- mediated ferroptosis	BALB/c nude mice (10, 30, 60 mg/kg)	HUH7 and HCCLM3 cells (20, 40, 60 µM)	[119]
Arenobufagin		Steroids	HCC	Inhibiting the p62‑Keap1‑Nrf2 pathway to induce autophagy‑dependent ferroptosis	BALB/c nude mice (1, 3, 6 mg/kg)	HepG2 cells (25, 50, 100 µM)	[120]
Picropodophyllin		Lignans	HCC	Blocking the AKT/ Nrf2/SLC7A11 and AKT/ Nrf2/SLC40A1 axes	BALB/c nude mice (25 mg/kg and 50 mg/kg)	Huh7 and PLC/PRF/5 cells (0.175, 0.35, 0.7 µM)	[121]
Curcumin		Polyphenols	HCC	Up-regulating ACSL4	Nude mice (20 mg/kg)	HepG2 and SMMC7721 cells (10, 20, 40 µM)	[122]
α-Hederin		Terpenoids	HCC	Inhibiting GPX4 and Xct, promoting DMT1	–	Bel-7402 (25, 50 µM)	[123]

### Context-dependent regulation of ferroptosis

This review mechanistically details multiple pathways that either promote or inhibit ferroptosis. However, the regulatory network governing ferroptosis exhibits significant paradoxical or context-dependent characteristics. This observation indicates that its biological effects are highly contingent upon cell type, stress context, levels of ROS, as well as the subcellular localization and duration of expression of regulatory factors. Among the diverse signaling pathways regulating ferroptosis, Nrf2 and its downstream effector, HO-1, constitute a pivotal axis that governs oxidative stress defense, iron metabolism, and ROS detoxification [125].

Nrf2, recognized as the primary regulator of antioxidant responses, typically enhances glutathione synthesis by upregulating the expression of GCLM and GCLC. Furthermore, it significantly suppresses ferroptosis through the direct transcriptional activation of SLC7A11 and GPX4. However, under certain conditions, sustained or excessive activation of Nrf2 may indirectly promote the accumulation of lipid peroxidation by upregulating genes with pro-oxidative potential, such as HO-1, or by altering iron metabolism through the regulation of ferritin heavy chain FTH1. Consequently, this may result in a pro-ferroptotic tendency [126, 127].

HO-1 exemplifies the complexity of ferroptosis regulation. Its products, bilirubin and carbon monoxide, exert antioxidant and anti-inflammatory effects that protect cells from ferroptosis [128]. However, the catalysis of heme degradation by HO-1 also results in the release of free iron. This release may exacerbate intracellular iron instability, promote Fenton reactions, and consequently enhance susceptibility to lipid peroxidation and ferroptosis. The net effect of HO-1 activity—whether it promotes or suppresses ferroptosis—depends on the overall balance of its expression levels, the duration of this expression, and the integrated iron homeostasis and antioxidant capacity of the cell [127, 129].

The protein p53 exhibits dual functions in the regulation of ferroptosis. On one hand, p53 promotes ferroptosis by transcriptionally suppressing the expression of SLC7A11, which reduces GSH levels and results in the accumulation of intracellular lipid peroxides [49]. Conversely, p53 can also inhibit ferroptosis by activating a range of antioxidant genes or by regulating cell cycle dynamics and metabolic reprogramming [130].

In summary, the dual roles exhibited by key factors such as Nrf2, p53, and HO-1 in ferroptosis suggest that they cannot be merely classified as simple "promoting" or "inhibiting" switches. Their functional outputs are significantly influenced by the specific cellular microenvironment and the pathological context. Therefore, understanding the intrinsic mechanisms and boundary conditions that govern the role transitions of these factors is essential for developing precise ferroptosis-targeting strategies, thereby minimizing the risk of unpredictable off-target effects that may arise from single-pathway interventions.

### Ferroptosis, TME, and immunomodulation

Research indicates that immune cells within the TME, such as dendritic cells, T cells, and macrophages, exhibit growth signaling and metabolic characteristics similar to those of cancer cells [64]. This functional overlap suggests that targeting tumor metabolism could simultaneously influence immune cell function. For example, ferroptosis inducers trigger lipid peroxidation in immune cells within the TME, which can impair their viability and function [131]. Activated T cells and dendritic cells depend on the glutathione system to sustain redox balance, while the polarization states of macrophages are closely associated with lipid metabolism [132]. Consequently, traditional ferroptosis-inducing strategies aimed at cancer cells may inadvertently compromise the function of anti-tumor immune cells, thereby diminishing the overall immune response.

In this context, the holistic perspective and therapeutic principle of "strengthening the body's defenses and eliminating pathogens" in TCM provide a distinctive approach to addressing these challenges. Numerous natural products derived from TCM have been validated as effective inducers of ferroptosis; however, their effects extend beyond mere "cell killing." Rather, they often involve complex immunomodulatory functions. The multi-component nature of TCM formulas presents both opportunities and challenges for modulating the TME. While individual compounds may exert specific effects on cancer cells—such as inducing ferroptosis—their impact on immune cell populations within the TME requires careful evaluation. For instance, certain natural products have been shown to influence macrophage polarization or T cell function, which could either synergize with or counteract anti-tumor immunity depending on the context [133, 134]. Therefore, a precise understanding of the cellular targets and context-dependent effects of these compounds is essential for developing effective combination strategies.

In liver cancer, oxymatrine effectively inhibits tumor growth, reduces IFN-γ secretion, and downregulates PD-L1 protein levels in tumor tissues. Additionally, it influences ferroptosis-related proteins, including xCT and GPX4, thereby promoting ferroptosis-mediated cell death. By mitigating IFN-γ-induced immune reprogramming and restoring T cell-mediated cytotoxicity, oxymatrine demonstrates significant immunomodulatory potential in liver cancer therapy [135]. In gastric cancer, asiaticoside significantly increases intracellular Fe^2^⁺ levels, reduces intracellular ROS levels, decreases GPX4 and SLC7A11 expression, and lowers GSH levels. Furthermore, it elevates the percentage of CD8 + T cells and IFN-γ concentration while simultaneously reducing PD-L1 expression and IL-10 content. In summary, these findings indicate that asiaticoside suppresses the malignant proliferation of gastric cancer cells by enhancing ferroptosis and inhibiting immune evasion [136]. Cetuximab is a commonly used epidermal growth factor receptor inhibitor, and developing resistance to it poses a major challenge in the clinical treatment of KRAS-mutated colorectal cancer. Zuojinwan, a traditional Chinese medicine formulation, demonstrates potential in reversing cetuximab resistance in KRAS-mutated colorectal cancer by targeting ferroptosis-related pathways [133].

A growing body of evidence highlights the critical role of natural products in regulating ferroptosis and enhancing immune responses across various malignancies. Despite their structural diversity, these products target similar molecular pathways, including GPX4, SLC7A11, and the IFN-γ/PD-L1 axis, indicating a shared mechanism of action. Not only do these products promote ferroptosis, but they also modulate the tumor immune microenvironment, thereby improving the efficacy of ICIs [65]. Their capability to simultaneously target immune evasion mechanisms and ferroptosis pathways underscores their potential as adjuvants in cancer immunotherapy [134]. However, existing research primarily emphasizes their antitumor effects, leaving unclear whether TCM-mediated regulation of ferroptosis may provoke pro-inflammatory responses in specific microenvironments, potentially facilitating tumor growth. Consequently, future studies must elucidate their molecular mechanisms and advance clinical translation while systematically evaluating potential risks to provide comprehensive evidence for the safe and effective implementation of combination therapies.

### Common mechanisms and cross-cancer targets

Through a systematic review of existing research, we found that, despite their diverse origins and chemical structures, these natural products share a common mechanism for inducing ferroptosis: they disrupt redox homeostasis and iron metabolism within cancer cells through synergistic actions that target multiple pathways [137]. First, numerous products, such as Polyphyllin B/I/VI, Baicalein, Emodin, and Curcumin, primarily weaken the core antioxidant defense system by downregulating the expression of SLC7A11 or GPX4, or by promoting their proteolytic degradation, which leads to the accumulation of lipid peroxides [92, 93, 104, 116, 117, 122]. Second, another class of products focuses on disrupting iron homeostasis by elevating unstable intracellular Fe^2^⁺ levels through various pathways. For instance, Salidroside, Gastrodin, and d-Borneol promote iron release via NCOA4-mediated ferritin autophagy [76, 79, 98], while Quercetin and Sinapine achieve this by increasing cellular iron uptake [75, 105]. Additionally, Arenobufagin intervenes in ferroptosis via the p62-Keap1-Nrf2 pathway, Erianin does so through the Ca^2^⁺/CaM signaling pathway, and Icariin induces mitochondrial dysfunction [70, 95, 120]. This diversity of action demonstrates the extensive coverage and profound intervention capabilities of natural products within the complex regulatory network of ferroptosis.

Despite the cancer-specific variations, certain core targets exhibit cross-cancer regulatory significance. As a principal executor of ferroptosis, the expression levels of GPX4 directly influence tumor sensitivity to ferroptosis [30, 138]. Research indicates that GPX4 can be targeted by various natural products derived from TCM, including Timosaponin AIII in lung cancer, Nobiletin in breast cancer, and Baicalein in colorectal cancer. Additionally, SLC7A11, a key subunit of System Xc⁻, is inhibited by Quercetin in gastric cancer, downregulated by α-Hederin in hepatocellular carcinoma, and regulated by Bufalin in breast cancer. These interventions effectively disrupt glutathione synthesis pathways [138]. In models of colorectal, liver, and breast cancer, gastrodin, esculetin, and salidroside demonstrate antitumor effects by activating the NCOA4-mediated ferritin autophagy pathway. This mechanism enhances lipid peroxidation through the promotion of ferritin degradation and iron ion release, thereby establishing a commonality in iron metabolism regulation across different cancer types [139]. The identification of these shared targets offers a clear direction for the development of broad-spectrum ferroptosis inducers derived from TCM.

Numerous natural products, including Ginkgetin, Dihydroartemisinin, Baicalin, and Icariin, demonstrate significant synergistic effects when used in conjunction with conventional chemotherapeutic agents such as cisplatin, 5-fluorouracil, and oxaliplatin, or with targeted therapies like PD-1 inhibitors. This evidence suggests that the induction of ferroptosis may serve as a promising strategy to overcome tumor resistance.

### Clinical translation challenges

Despite the potential of clinical translation, several challenges persist. Firstly, the druggability of natural products presents a pressing concern. Many natural products exhibit poor water solubility, low bioavailability, and metabolic instability, which necessitate structural modifications and formulation optimizations, such as the development of nanodelivery systems, to improve their pharmacokinetic properties [140, 141]. Nano-drug delivery systems utilize functionalized nanocarriers for targeted drug delivery. Drugs can adsorb onto their surfaces or be encapsulated within them, facilitating targeted delivery to specific sites. This approach enhances solubility and stability, improves absorption, and allows for controlled release. In one study [142], a nano-precipitation method was employed to prepare nano-structured liposomes coated with hyaluronic acid, featuring gemcitabine and baicalin as core components—hyaluronic acid-Gemcitabine-Baicalin Nanostructured Liposomes. The combination of baicalin and gemcitabine effectively overcomes multidrug resistance and mitigates adverse reactions during treatment. The nanoliposomes prolong the plasma half-life of the drugs and enhance their antitumor activity. These nanostructured liposomes can penetrate pancreatic tumor cells, exhibiting cytotoxicity and inhibiting their growth. Another research effort successfully developed ethylene glycol chitosan nanoparticles co-loaded with gambogic acid and retinoic acid chlorochalcone [143]. Both gambogic acid and retinoic acid chlorochalcone effectively inhibit tumor growth by suppressing tumor cell proliferation and inducing differentiation. The high permeability and retention properties of the nanoparticles enable efficient accumulation of gambogic acid and retinoic acid chlorochalcone within tumors. Additionally, research has synthesized resveratrol-gold nanoparticles (Res-CNPs), which significantly increased tumor necrosis areas compared to free resveratrol drugs [144]. Res-CNPs markedly inhibited the proliferation of hepatocellular carcinoma without causing notable toxicity to major organs, thus offering a novel approach for the safe and efficient delivery of natural products with ferroptosis-inducing potential. In recent years, TCM‑based nanomedicines have been widely investigated in preclinical research and are gradually entering clinical trials. These nanomedicines play a significant role in prolonging the duration of drug action, enhancing bioavailability, improving pharmacokinetics, and reducing drug toxicity, thereby demonstrating promising therapeutic outcomes and broad prospects. Consequently, the deep integration of active components from TCM with modern nanotechnology has emerged as an effective strategy to overcome drug development bottlenecks, achieve clinical translation, and enhance therapeutic efficacy. Secondly, the complex composition of TCM demands standardized quality control for its active ingredients [145]. Safety is a critical aspect of quality research in TCM. The complexity of toxic substances, the unclear boundaries between toxicity and efficacy, the unknown mechanisms of toxicity and mitigation, the ambiguous safe dosages, and the lack of precise detection technologies for systemic harmful residues constitute significant bottlenecks in ensuring the safe use of TCM. These challenges constrain the modern development and international expansion of the TCM industry. Future efforts should focus on elucidating the mechanisms of toxicity and detoxification principles, establishing evaluation systems for safe dosages and toxic components of TCM, and developing precise detection technologies for harmful residues. Implementing these measures will ensure medication safety and promote the sustainable development and international expansion of TCM. Furthermore, the specific molecular targets of numerous products remain unidentified, highlighting the need for comprehensive exploration through chemical biology approaches [146]. Additionally, preclinical studies predominantly rely on cell and animal models, lacking substantial human clinical data. The differential regulation of ferroptosis in various cancers may also contribute to treatment response heterogeneity, emphasizing the urgent requirement for precision-oriented clinical research. A noteworthy double-blind trial has demonstrated that curcumin and nano-curcumin significantly outperform placebo in their anti-inflammatory and antioxidant effects [147]. Similarly, a study on sepsis revealed that curcumin reduces serum inflammatory markers (IL-6, IL-18) and oxidative stress indicators, such as MDA, while simultaneously increasing antioxidants like Nrf2, catalase, and superoxide dismutase. These results suggest that curcumin has the potential to regulate lipid peroxidation, which is closely related to ferroptosis. However, clinical trials in cancer patients are still needed to confirm its role as a ferroptosis inducer [148]. Furthermore, artemisinin has been shown to induce ferroptosis in tumor cells and has progressed to Phase I clinical trials, with completed studies indicating favorable safety and tolerability in patients.

## Future directions

To fully realize the clinical potential of natural products in modulating ferroptosis, future research should focus on several key areas. First, the field must progress from merely cataloging natural products towards mechanistic deepening and structural optimization. A dedicated effort to investigate the structure–activity relationships (SAR) of these agents is urgently needed. Future studies should focus on identifying the essential pharmacophores for ferroptosis induction and employing chemical modifications to enhance efficacy, reduce off-target effects, and improve drug-like properties. Second, as research on single-agent TCM-derived products advances, the critical next step is to address their clinical translation. Key questions must be answered: How can we rationally design clinical trials to evaluate the synergy between TCM-derived ferroptosis inducers (including their optimized formulations) and standard therapies such as chemotherapy, radiotherapy, or immune checkpoint inhibitors? Furthermore, what reliable biomarkers, such as those derived from lipid peroxidation, GPX4 activity, or iron metabolism, can be developed to predict and monitor patient responses to these therapies? Third, while research on single agents is progressing, investigations into multi-component TCM formulas capable of inducing tumor ferroptosis remain limited. This gap may stem from the complexity of tumor pathogenesis and the current lack of well-defined, ferroptosis-targeting formulas. Future exploration in this area could be significantly informed by the accumulated clinical experience of renowned senior TCM practitioners, offering a unique systems-based approach to modulating ferroptosis.

## Conclusions

In summary, TCM and its natural products exhibit great potential and unique characteristics in cancer therapy, representing a valuable source of ferroptosis inducers. Their multi-component and multi-target characteristics align exceptionally well with the complex regulatory network of ferroptosis, demonstrating significant potential in the field of targeted ferroptosis-based anticancer therapy. By elucidating their mechanisms of action through modern scientific techniques, along with drug delivery optimization and precision clinical research, these therapies promise to deliver novel cancer treatments that integrate the distinctive features of TCM with contemporary scientific evidence.

## Data Availability

Not applicable.

## Abbreviations

ACSL4: Acyl-CoA synthetase long-chain family member 4
BH4: Tetrahydrobiopterin
CO: Carbon monoxide
COQ10: Coenzyme Q10
COQ10H2: Coenzyme Q10 Hydroquinone
DHODH: Dihydroorotate dehydrogenase
FPN: Ferroportin
FSP1: Ferroptosis suppressor protein 1
FTH1: Ferritin Heavy Chain 1
FTL: Ferritin Light Chain
GCH1: GTP cyclohydrolase 1
GCLC: Glutamate-Cysteine ligase catalytic subunit
GCLM: Glutamate-Cysteine ligase modifier subunit
Glu: Glutamate
GPX4: Glutathione peroxidase 4
GSH: Glutathione
GSSG: Glutathione Disulfide
GTP: Guanosine triphosphate
HO-1: Heme oxygenase-1
HSPB1: Heat shock protein family member B-1
ICIs: Immune checkpoint inhibitors
KEAP1: Kelch-Like ECH-Associated protein 1
LIP: Labile iron pool
LOXs: Lipoxygenases
MUFAs: Monounsaturated fatty acids
NAD(P)H: Nicotinamide adenine dinucleotide (phosphate) hydride
NCOA4: Nuclear Receptor Coactivator 4
Nrf2: Nuclear factor E2-related factor 2
POR: Cytochrome P450 reductases
PUFAs: Polyunsaturated fatty acids
PUFA-PLOOH: Polyunsaturated fatty acid phospholipid hydroperoxides
ROS: Reactive oxygen species
SAT1: Spermidine/Spermine N1-Acetyltransferase 1
SCD1: Stearoyl-CoA desaturase 1
SLC3A2: Solute carrier family 3 member 2
SLC7A11: Solute carrier family 7 member 11
System Xc⁻: Cystine-glutamate antiporter system
TAMs: Tumor-associated macrophages
TCM: Traditional Chinese medicine
TF: Transferrin
TFR1: Transferrin receptor 1
TME: Tumor Microenvironment
